# Anaerobic Bioconversion of Mixed Fruit Waste into Organic Acids and a Multifunctional Enzymatic Bioproduct in a Stirred-Tank Bioreactor Using *Wickerhamomyces* sp. UFFS-CE-3.1.2

**DOI:** 10.3390/microorganisms14040907

**Published:** 2026-04-17

**Authors:** Vitória Dassoler Longo, Nair Mirely Freire Pinheiro Silveira, Marcelli Powzum Amorim, Emanuely Fagundes da Silva, Isabely Sandi Baldasso, Arielle Cristina Fornari, Mateus Torres Nazari, Sérgio L. Alves, Helen Treichel

**Affiliations:** 1 Laboratory of Microbiology and Bioprocess, Federal University of Fronteira Sul, Erechim 99700-000, RS, Brazil; vitorialongo22@gmail.com (V.D.L.); nairfreire460@gmail.com (N.M.F.P.S.); marcellipowzum@gmail.com (M.P.A.); emanuelyfagundesdasilva@gmail.com (E.F.d.S.); isabelysandibaldasso@gmail.com (I.S.B.); arielle.fornari@uffs.edu.br (A.C.F.); 2 Graduate Program in Civil and Environmental Engineering (PPGEng), University of Passo Fundo (UPF), Passo Fundo 99052-900, RS, Brazil; nazari.eas@gmail.com; 3 Laboratory of Yeast Biochemistry, Federal University of Fronteira Sul, Chapecó 89815-899, SC, Brazil; slalvesjr@uffs.edu.br

**Keywords:** fruit waste valorization, anaerobic fermentation, yeast, oxidative stress enzymes, circular bioeconomy

## Abstract

The microbial valorization of agro-industrial residues is a promising strategy for sustainable bioprocesses and the development of a circular bioeconomy. In this study, mixed fruit peel waste was anaerobically fermented in a stirred-tank bioreactor using *Wickerhamomyces* sp. UFFS-CE-3.1.2 to produce organic acids and a multifunctional enzymatic bioproduct. During fermentation, sugars decreased from 6.51 to 0.22 g L^−1^, leading to the formation of citric acid (7.65 g L^−1^), ethanol (3.77 g L^−1^), glycerol (0.53 g L^−1^), and acetic acid (0.37 g L^−1^). The accumulation of organic acids likely imposed metabolic stress on the yeast, triggering physiological responses that mitigate oxidative stress. Consequently, the resulting enzymatic extract exhibited high lipase activity (185.63 U mL^−1^), late catalase induction (520.97 U mL^−1^), and stable superoxide dismutase activity (50 U mL^−1^). This enzymatic profile indicates the formation of a stress-adapted microbial system with potential applicability in processes involving lipid hydrolysis and oxidative mechanisms. The process was conducted without supplementation of synthetic medium and operated stably in a stirred-tank bioreactor. Overall, these results suggest a feasible microbial strategy for converting fruit waste into value-added bioproducts, contributing to the development of sustainable biotechnological processes.

## 1. Introduction

The increase in food waste generation has intensified in parallel with global population growth, becoming one of the main environmental challenges of recent decades [1]. The world currently hosts nearly 9 billion people, whose patterns of food consumption and disposal are continuously accelerating [2,3]. In response to this scenario, food waste reduction has been established as a priority within the United Nations Sustainable Development Goals (SDGs), which propose a 50% reduction in global per capita food waste at the retail and consumer levels by 2030, as well as a significant decrease in post-harvest and supply chain losses [4].

The improper disposal of food residues leads to overloading of landfills, open dumping areas, soils, and wastewater systems, promoting the proliferation of pathogenic microorganisms and causing adverse impacts on both the environment and public health [5]. Among food residues, fruit waste represents a major fraction due to its large-scale consumption and processing, with global estimates ranging from 25 to 57 million tons per year [6]. The magnitude of this issue is evident from reports, such as the Indian Ministry of Food Processing Industries’ report, which estimates that approximately 12 million tons of fruit-processing residues are generated annually [7]. When properly valorized, these residues can be converted into high-value bioproducts, contributing to circular economy strategies and environmentally sustainable production systems.

Anaerobic fermentation has emerged as a viable alternative for the reuse of organic residues while simultaneously mitigating carbon emissions [8]. From an operational perspective, anaerobic systems offer significant advantages, including simpler reactor configurations, lower energy requirements, reduced aeration costs, and greater resistance to oxygen-transfer limitations [9]. Despite promising results at the laboratory scale, the transition to larger-scale systems remains a major technological challenge in bioprocess development. Scale-up affects key parameters, including mixing efficiency, mass transfer, microbial stability, and economic feasibility [10]. In this context, anaerobic biotechnological routes stand out for their operational robustness, lower energy demand, and greater tolerance to process fluctuations, characteristics that favor their application at pilot and industrial scales [11]. The use of agro-industrial residues as substrates further improves process feasibility by significantly reducing raw material costs.

Fruit peels are rich in bioactive compounds, including phenolics, carotenoids, vitamins, organic acids, essential oils, and enzymes, which makes them attractive feedstocks for biotechnological applications [12]. In addition, their high content of fermentable sugars enables their use in microbial processes for the production of metabolites of industrial interest [13,14]. Citrus residues, particularly orange and lemon peels, have received special attention due to their potential to recover high-value compounds within biorefinery concepts [15,16]. In recent years, the concept of biorefineries has gained increasing attention as a sustainable strategy for the integrated valorization of biomass. Within this framework, agro-industrial residues are not treated as waste but as renewable feedstocks for the simultaneous production of multiple value-added products, including biofuels, organic acids, and enzymes. This approach maximizes resource efficiency and aligns with circular bioeconomy principles by reducing waste generation while increasing the economic value of biomass streams. In this context, the use of heterogeneous substrates, such as mixed fruit residues, represents a promising yet underexplored opportunity. Their complex composition and variability pose challenges but also create opportunities for generating multifunctional bioproducts.

Yeasts of the genus *Wickerhamomyces* have attracted increasing attention in recent years due to their metabolic versatility and their ability to adapt to complex substrates. These microorganisms can utilize a wide range of carbon sources and have been associated with the production of organic acids, alcohols, and various enzymes under different cultivation conditions. In addition, their tolerance to environmental stress factors, such as low pH and the presence of inhibitory compounds, makes them suitable candidates for the bioconversion of agro-industrial residues. However, their behavior under anaerobic conditions in stirred-tank bioreactors, particularly regarding the simultaneous production of metabolites and production of multifunctional enzymatic systems, remains insufficiently explored. Despite these advances, most studies on fruit waste bioconversion have focused on the production of single metabolites or on enzyme synthesis under aerobic conditions in chemically defined media. Investigations into the generation of multifunctional enzymatic extracts from mixed fruit residues remain limited, particularly under anaerobic conditions and in mechanically stirred bioreactors, where substrate heterogeneity, mass transfer, and microbial physiology play a decisive role in process performance. Moreover, although *Wickerhamomyces* spp. have demonstrated metabolic versatility and tolerance to complex substrates, their potential for the simultaneous production of oxidative stress-related and hydrolytic enzymes using agro-industrial waste as the sole carbon source remains poorly explored. Therefore, the development of anaerobic bioprocesses capable of converting heterogeneous fruit residues into multifunctional enzymatic bioproducts represents an important scientific and technological gap.

In this context, the present study aimed to evaluate metabolite formation and enzymatic activities during anaerobic fermentation of mixed fruit peel waste in a stirred-tank bioreactor using *Wickerhamomyces* sp. UFFS-CE-3.1.2. Rather than targeting the production of a single metabolite, the study focuses on generating a multifunctional enzymatic bioproduct under bioreactor conditions.

## 2. Materials and Methods

### 2.1. Biomass

The biomass used in this study consisted of orange, banana, mango, pineapple, and lemon peels collected from the university restaurant. The residues were stored separately at −60 °C until sufficient amounts were obtained. Subsequently, the material was dried in a forced-air circulation oven at 40 °C for 48 h and milled in a knife mill to a 20-mesh particle size [17]. The moisture content was determined using a moisture analyzer in order to standardize the biomass prior to its use in the fermentation assays [18]. The selected fruit residues were chosen due to their availability and representativeness of typical agro-industrial waste generated in food service environments. The use of mixed fruit peels allows the simulation of real waste streams, which are inherently heterogeneous in composition. This characteristic is important for evaluating the robustness of the fermentation process under conditions that better reflect practical applications.

### 2.2. Biomass Washing and Soluble Sugar Extraction

Soluble sugars were extracted from the biomass by washing 46.2 g of the mixed dried peels (9.24 g of each fruit) with 500 mL of 0.05 M sodium citrate buffer (pH 4.8). This condition was selected based on a previously optimized protocol for the same fruit mixture at a smaller scale [17]. The washing step was designed to recover soluble sugars while minimizing insoluble solids, thereby enabling better control of fermentation conditions and facilitating subsequent analytical determinations.

The composition of the fruit mixture was based on a previously optimized ratio for bioethanol production [17]. In the present study, this mixture was not intended for enzyme optimization but rather used as a standardized, reproducible substrate to evaluate microbial metabolic behavior and enzymatic activity under anaerobic bioreactor conditions. The initial soluble sugar concentration of the obtained liquid fraction was 6.51 g L^−1^, and the initial pH was 4.8.

### 2.3. Inoculum Preparation

The strain *Wickerhamomyces* sp. UFFS-CE-3.1.2 was previously isolated and identified by our group [19] and is maintained in the microbial culture collection of the Federal University of Fronteira Sul. The strain was reactivated on solid YPD medium composed of yeast extract (1%), peptone (2%), glucose (2%), and agar (2%). After 72 h of incubation, the cells were transferred to liquid YPD medium (without agar) and incubated for 24 h in a BOD incubator to allow adaptation prior to fermentation. The inoculum was standardized to approximately 1 × 10^6^ CFU mL^−1^ and added to the fermentation medium at 10% (*v*/*v*) [20]. The pre-cultivation step was performed to ensure metabolic activation of the cells and to promote adaptation to the fermentation conditions prior to inoculation.

### 2.4. Anaerobic Fermentation in a Stirred-Tank Bioreactor

The liquid fraction obtained from biomass washing was transferred to a mechanically stirred bioreactor (Tecnal, Piracicaba, Brazil) with a working volume of 1.2 L and sterilized at 121 °C for 20 min. After cooling to 30 °C, the medium was inoculated with 10% (*v*/*v*). The use of a stirred-tank bioreactor allows improved control of mixing and environmental conditions, enabling a more homogeneous distribution of nutrients and microbial cells. The absence of aeration was intended to simulate strict anaerobic conditions, which are relevant for the production of organic acids and the evaluation of stress-related enzymatic responses.

Fermentation was conducted at 30 °C under constant agitation at 150 rpm. Anaerobic conditions were established by maintaining the reactor closed, without aeration. Dissolved oxygen (DO) was monitored with an electrode, confirming the establishment of anaerobic conditions approximately 30 min into fermentation.

The pH was not controlled during the process. Samples were collected at 0, 18, 24, 48, and 72 h for analytical determinations.

All fermentation experiments were performed in triplicate (three independent biological replicates).

### 2.5. Enzymatic Activity Assays

Enzymatic activities of environmental interest were determined according to the methodologies described by Longo et al. [21]. The selection of enzymes analyzed in this study was based on their relevance to environmental and industrial applications, particularly those associated with oxidative stress response and substrate hydrolysis.

#### 2.5.1. Amylase and Cellulase

Both activities were quantified by measuring total reducing sugars using the 3,5-dinitrosalicylic acid (DNS) method at 540 nm [22]. For amylase activity, the enzymatic extract was incubated with soluble starch (1:100, *m*/*v*) in 100 mM acetate buffer (pH 5.0) at 38 °C for 10 min [23,24]. For cellulase activity, 50 mg of Whatman No. 1 filter paper was incubated with 2 mL of buffer and 1 mL of enzymatic extract at 50 °C for 1 h [25]. Enzyme activities were calculated from a glucose standard curve and expressed as U mL^−1^.

#### 2.5.2. Laccase

Laccase activity was determined using 2,2′-azino-bis(3-ethylbenzothiazoline-6-sulfonic acid) (ABTS) as substrate. The reaction mixture contained 0.4 mL of 0.01 M ABTS, 0.2 mL of fermented extract, and 3.4 mL of buffer. After 4 min of incubation, absorbance was measured at 420 nm. One unit of enzyme activity was defined as the amount of enzyme required to oxidize 1 µmol of ABTS per minute under the assay conditions [26].

#### 2.5.3. Lipase

Lipase activity was determined according to Treichel et al. [27] using an emulsion containing 10% (*m*/*v*) olive oil and 5% (*m*/*v*) gum arabic prepared in buffer (90%, *v*/*v*). The reaction mixture consisted of 1 mL of enzymatic extract and 9 mL of substrate, incubated at 165 rpm for 32 min. The reaction was stopped by adding 10 mL of acetone-ethanol solution, followed by titration with 0.049 M NaOH up to pH 11. One unit of lipase activity corresponded to the amount of enzyme that released 1 µmol of fatty acid per minute under the assay conditions.

#### 2.5.4. Protease

Protease activity was determined using casein as the substrate, according to the modified method of Waghmare et al. [28]. The reaction mixture containing casein, enzymatic extract, and buffer was incubated for 30 min in a thermostatic bath. The reaction was stopped with trichloroacetic acid, and the supernatant was then treated with a sodium carbonate buffer and the Folin reagent. Absorbance was measured at 660 nm. One unit of activity was defined as the amount of enzyme required to release 1 µg of tyrosine per minute.

#### 2.5.5. Peroxidase

The reaction mixture consisted of buffer, distilled water, 1% guaiacol, hydrogen peroxide (8%), and enzymatic extract. After 20 min of incubation, absorbance was measured at 470 nm. One unit of peroxidase activity corresponded to an increase of 0.001 in absorbance per minute [29,30].

#### 2.5.6. Catalase

Catalase activity was determined according to Havir and McHale [31] and Hasan et al. [32]. The reaction mixture contained buffer, distilled water, enzymatic extract, and 0.0125 M hydrogen peroxide. The decrease in absorbance was monitored at 240 nm every 30 s for 3 min.

#### 2.5.7. Ascorbate Peroxidase

The assay was performed according to the methods of Nakano and Asada [33] and Fal et al. [34]. The reaction mixture contained buffer, distilled water, 0.008 M ascorbic acid, enzymatic extract, and 0.001 M hydrogen peroxide. Absorbance was measured at 290 nm for 1 min, with readings taken every 15 s.

#### 2.5.8. Superoxide Dismutase

Superoxide dismutase activity was determined as described by Hasan et al. [32]. The reaction mixture contained buffer, methionine, nitroblue tetrazolium (NBT), EDTA, riboflavin, distilled water, and enzymatic extract. The samples were exposed to a 15 W fluorescent lamp, and absorbance was measured at 560 nm. The control was kept in the dark. One unit of SOD activity was defined as the amount of enzyme required to inhibit 50% of NBT photoreduction.

### 2.6. High-Performance Liquid Chromatography (HPLC)

HPLC analyses were performed to quantify glucose, cellobiose, arabinose, and fructose, as well as ethanol, citric acid, and acetic acid produced during fermentation. The use of HPLC enabled accurate quantification of both substrate consumption and metabolite production, allowing the evaluation of fermentation performance over time. Samples were previously diluted in 0.005 M sulfuric acid, filtered through a Millipore membrane, and degassed in an ultrasonic bath for 15 min [20]. The chromatographic system (Shimadzu, Kyoto, Japan) was equipped with a refractive index detector and an Aminex Bio-Rad HPX-87H (Bio-Rad Laboratories, Hercules, CA, USA) column. The analyses were carried out at 45 °C with a flow rate of 0.6 mL min^−1^ [19].

### 2.7. Statistical Analysis

All results are presented as mean values ± standard deviation of triplicate experiments. Statistical differences between samples were evaluated using analysis of variance (ANOVA), followed by Tukey’s post hoc test when applicable. Differences were considered statistically significant at *p* < 0.05.

## 3. Results and Discussion

### 3.1. Carbon Conversion and Metabolite Distribution During Anaerobic Bioconversion of Fruit Waste

The anaerobic fermentation of the soluble fraction obtained from mixed fruit peels produced citric acid, glycerol, acetic acid, and ethanol, demonstrating the effective microbial conversion of this agro-industrial residue into value-added metabolites. The total sugar concentration decreased from 6.51 to 0.22 g L^−1^ within 48 h, indicating efficient substrate consumption and confirming the suitability of the biomass as a fermentable carbon source.

Citric acid was the main metabolite formed, increasing from 3.18 to 7.65 g L^−1^ after 48 h. This behavior is consistent with previous studies reporting citric acid production in the range of 5–10 g L^−1^ during anaerobic fermentation of agro-residues under stress conditions [35], in which organic acid accumulation is favored over ethanol production. Acetic acid and glycerol were also produced, reaching 0.37 and 0.53 g L^−1^, respectively, while ethanol reached a maximum of 3.77 g L^−1^.

Compared with previous small-scale experiments using the same substrate, the ethanol concentration obtained in the stirred-tank bioreactor was lower (7.14 g L^−1^ in flask cultivation), indicating a clear scale effect on carbon flux distribution. In complex media derived from lignocellulosic and fruit residues, nutrient and metabolite gradients can alter microbial metabolism and product formation [36]. In addition, the accumulation of undissociated acetic acid can impair sugar uptake and reduce fermentative efficiency due to intracellular acidification [37].

From a waste valorization perspective, this metabolic shift should not be interpreted as a process limitation but rather as a redirection of carbon toward the generation of organic acids and a functionally enriched fermentation broth. Organic acids themselves are value-added products and, more importantly, their accumulation promoted the formation of a stress-adapted enzymatic system with potential applicability, which should be further evaluated in future studies.

### 3.2. Acidification as a Driver for the Formation of a Functionally Specialized Enzymatic Extract

The progressive accumulation of citric and acetic acids likely reduced the medium pH, creating an acidic environment that affected yeast metabolism. Acidic stress is known to reduce ethanol yield while activating cellular defense mechanisms [38,39]. In the present study, this condition appears to have played a central role in defining the functional profile of the enzymatic extract.

Instead of favoring a classical hydrolytic enzyme-rich system, the anaerobic reactor environment promoted the formation of an enzymatic profile associated with oxidative stress response. This shift is relevant from a biotechnological perspective, as enzymes involved in reactive oxygen species detoxification are commonly associated with oxidative metabolic pathways.

However, it is important to note that applications such as contaminant degradation, oxidative bioremediation, or bioherbicidal activity were not directly evaluated in this study. Therefore, the observed enzymatic profile should be interpreted as indicative of potential functionality and warrant further investigation in future studies.

Thus, the operational conditions of the anaerobic stirred-tank reactor served not only as a conversion platform for waste but also as a selective pressure that influenced the biochemical functionality of the resulting bioproduct.

### 3.3. Enzymatic Consortium Generated from Fruit Waste and Its Environmental Relevance

The enzymatic profile obtained during fermentation (Table 1) demonstrated the formation of a multifunctional extract with distinct catalytic potential. The temporal evolution of metabolites and enzymatic activities is clearly reflected in the data presented in Table 1, allowing direct comparison of trends throughout the fermentation process.

Lipase activity showed the most pronounced increase, reaching 185.63 U mL^−1^ at 48 h, while it was absent in the initial medium. This value was higher than those reported for yeast lipase production in defined media [40] and also higher than values obtained for Antarctic yeasts cultivated in lipid-based substrates [41], demonstrating the effectiveness of fruit residues as low-cost substrates for enzyme production. From a biotechnological perspective, lipases are widely known for hydrolyzing lipid-rich substrates.

Catalase showed a late but expressive induction, reaching 520.97 U mL^−1^ at 72 h. In combination with stable superoxide dismutase activity (50 U mL^−1^), this result indicates the establishment of an oxidative stress response system. These enzymes are recognized for their role in protecting cells against reactive oxygen species [42,43].

In contrast, cellulase and amylase activities decreased throughout fermentation, indicating depletion of structural substrates and/or destabilization of the enzymes. Peroxidase and ascorbate peroxidase also showed reduced activity, suggesting that the process conditions selectively favored enzymes associated with oxidative stress response rather than lignocellulose hydrolysis. Laccase activity was not detected, which is consistent with the absence of specific inducers in the medium [26]. Protease activity remained relatively stable and within the range reported for other yeast systems [44], reinforcing the ability of *Wickerhamomyces* sp. UFFS-CE-3.1.2 to produce multiple enzymes using complex residues as the sole carbon source.

From a circular bioeconomy perspective, these results demonstrate that mixed fruit waste can be converted into a functionally specialized enzymatic extract without supplementation with synthetic media. The anaerobic stirred-tank system served as both a conversion and selection platform, enabling the generation of an enzyme-rich extract with potential applicability that warrants further evaluation in future studies.

### 3.4. Implications for Fruit Waste Valorization and Environmental Biotechnology

The present study demonstrates that anaerobic fermentation of mixed fruit residues can be used to generate a multifunctional enzymatic extract rather than a single target metabolite. This approach aligns with the biorefinery concept, which aims to maximize the functional value of the resulting bioproduct.

The formation of an enzymatic system enriched in lipase, catalase, and superoxide dismutase suggests potential applicability in processes involving lipid hydrolysis and oxidative mechanisms. However, these applications were not directly evaluated in this study and should be considered as perspectives for future research.

In addition, successful operation in a stirred-tank bioreactor demonstrates the process’s feasibility under controlled conditions. Further studies are required to assess scalability and industrial applicability.

## 4. Conclusions

This study demonstrates the potential of mixed fruit peel waste as a low-cost substrate for microbial bioprocesses, highlighting an effective strategy for valorizing agro-industrial residues within a circular bioeconomy framework. Anaerobic fermentation conducted in a stirred-tank bioreactor using *Wickerhamomyces* sp. UFFS-CE-3.1.2 enabled the conversion of fruit waste into multiple value-added products, including organic acids, ethanol, and a multifunctional enzymatic extract.

The metabolic profile observed during fermentation, particularly the production of citric acid alongside ethanol and glycerol, supports the potential of this process to generate relevant metabolites from renewable substrates. In addition, the enzymatic extract exhibited significant lipase and oxidative stress-related enzyme activities, suggesting potential applicability in processes involving lipid hydrolysis and oxidative mechanisms. However, these applications were not directly evaluated in this study and require further investigation.

Importantly, the process was carried out without synthetic medium supplementation and operated stably in a stirred-tank bioreactor. Nevertheless, further studies are required to evaluate scalability and industrial feasibility. Overall, these findings contribute to the development of strategies to transform fruit processing residues into value-added bioproducts, thereby supporting sustainable biotechnological processes.

## Figures and Tables

**Table 1:** Enzymatic activities (U mL^−1^) during anaerobic fermentation of mixed fruit waste by *Wickerhamomyces* sp. UFFS-CE-3.1.2 in a stirred-tank bioreactor.

Enzyme	Initial Medium	0 h	24 h	48 h	72 h
Lipase	0.00 ± 0.00 ᵉ	20.63 ± 0.93 ᵈ	88.13 ± 1.12 ^c^	185.63 ± 2.37 ᵃ	69.38 ± 0.85 ᵇ
Peroxidase	88.12 ± 2.02 ᵃ	71.25 ± 0.54 ᵇ	56.87 ± 0.89 ^c^	44.37 ± 1.37 ᵈ	61.25 ± 0.84 ^c^
Laccase	0.00 ± 0.00 ᵃ	0.00 ± 0.00 ᵃ	0.00 ± 0.00 ᵃ	0.00 ± 0.00 ᵃ	0.01 ± 0.01 ᵃ
Protease	51.94 ± 0.23 ᵃ	38.06 ± 0.56 ^c^	45.28 ± 0.89 ᵇ	38.05 ± 0.78 ^c^	40.56 ± 0.96 ^c^
Catalase	0.00 ± 0.00 ᵇ	0.00 ± 0.00 ᵇ	0.00 ± 0.00 ᵇ	0.00 ± 0.00 ᵇ	520.97 ± 5.25 ᵃ
Cellulase	7.10 ± 0.12 ᵃ	4.38 ± 0.15 ᵇ	0.30 ± 0.02 ^c^	0.31 ± 0.05 ^c^	0.37 ± 0.07 ^c^
Amylase	11.98 ± 1.01 ᵃ	5.32 ± 0.22 ᵇ	0.14 ± 0.01 ^c^	0.14 ± 0.01 ^c^	0.14 ± 0.02 ^c^
Ascorbate peroxidase	2.36 ± 0.79 ᵃ	2.30 ± 0.80 ᵃ	2.11 ± 0.90 ᵃ	2.07 ± 0.76 ᵃ	0.00 ± 0.00 ᵇ
Superoxide dismutase	0.00 ± 0.00 ᵇ	0.00 ± 0.00 ᵇ	50.00 ± 2.34 ᵃ	50.00 ± 2.45 ᵃ	50.00 ± 2.37 ᵃ

Data are presented as mean ± standard deviation (n = 3). Different letters in the same row indicate statistically significant differences (*p* < 0.05) as determined by ANOVA followed by Tukey’s test.

## Data Availability

The original contributions presented in this study are included in the article. Further inquiries can be directed to the corresponding author.
